# Natural Products as Leads in Schistosome Drug Discovery

**DOI:** 10.3390/molecules20021872

**Published:** 2015-01-23

**Authors:** Bruno J. Neves, Carolina H. Andrade, Pedro V. L. Cravo

**Affiliations:** 1 LabMol—Laboratory for Drug Design and Molecular Modeling, Faculdade de Farmácia, Universidade Federal de Goiás, Goiânia 74605-170, Brazil; E-Mail: carolina@ufg.br; 2 GenoBio—Laboratory of Genomics and Biotechnology, Instituto de Patologia Tropical e Saúde Pública, Universidade Federal de Goiás, Goiânia 74605-050, Brazil; E-Mail: pedrovcravo@gmail.com

**Keywords:** schistosomiasis, natural products, leads, chemical scaffolds, virtual screening

## Abstract

Schistosomiasis is a neglected parasitic tropical disease that claims around 200,000 human lives every year. Praziquantel (PZQ), the only drug recommended by the World Health Organization for the treatment and control of human schistosomiasis, is now facing the threat of drug resistance, indicating the urgent need for new effective compounds to treat this disease. Therefore, globally, there is renewed interest in natural products (NPs) as a starting point for drug discovery and development for schistosomiasis. Recent advances in genomics, proteomics, bioinformatics, and cheminformatics have brought about unprecedented opportunities for the rapid and more cost-effective discovery of new bioactive compounds against neglected tropical diseases. This review highlights the main contributions that NP drug discovery and development have made in the treatment of schistosomiasis and it discusses how integration with virtual screening (VS) strategies may contribute to accelerating the development of new schistosomidal leads, especially through the identification of unexplored, biologically active chemical scaffolds and structural optimization of NPs with previously established activity.

## 1. Introduction

Schistosomiasis is a neglected tropical disease caused by parasitic flatworms of the genus *Schistosoma*, with three species (*S. mansoni*, *S. haematobium*, and *S. japonicum*) accounting for the majority of human infections. These parasites cause a chronic and often debilitating infection that impairs development and productivity, and exposure to these parasites is strongly linked to extreme poverty [1]. Recent estimates of the World Health Organization suggest that more than 249 million people have been infected in 78 endemic countries located in sub-Saharan Africa, the Middle East, the Caribbean, and South America resulting in approximately 200,000 deaths annually [2].

Schistosomes have complex life cycles that involve vertebrate hosts (often a mammal) and invertebrate hosts (fresh water snails). Humans are usually infected by the penetration of cercariae through the skin when they come into contact with contaminated freshwater. Following penetration into human skin, the maturing larvae (schistosomules) require about 5–7 weeks before becoming adults and producing eggs. The eggs are either shed into the environment through feces or urine (*S. haematobium*) or are retained in host tissues where they induce inflammation and then die. The eggs that reach freshwater will hatch, releasing free-living ciliated miracidia that then infect a suitable snail host. In the snail, the parasite undergoes asexual replication through mother and daughter sporocyst stages, eventually shedding tens of thousands of cercariae into the water, which are able to cause new infections in humans [3,4].

Poor knowledge about the disease, poor sanitation, and a lack of effective health policies promote the dissemination of schistosomiasis in endemic countries. In the absence of an effective vaccine, the control of schistosomiasis relies on a single drug, praziquantel (PZQ), which has been used in mass drug administration programs since the 1970s [5]. PZQ offers high efficacy, excellent tolerability, few and transient side effects, oral administration, and a competitive cost [6]; it has also been shown to exhibit anti-fibrotic activity in mice infected with schistosomes [7,8]. Even without these specific concerns, reliance on a single drug for a disease affecting 249 million people is ill-advised. The occurrence of PZQ resistance in the field [9,10,11] or in the laboratory [12,13,14] has been described in several studies. Moreover, PZQ has sub-optimal efficacy against immature worms that are present in newly acquired infections [5]. Thus, new schistosomicidal drugs are urgently needed.

In this context, natural products (NPs) are structurally diverse and serve as a valuable source for novel molecular scaffolds in drug development. The term “molecular scaffold” is used to describe the core structure of a molecule [15]. Around 65% of all approved drugs are classified as NPs or are inspired by an NP core [16]. Therefore, it is believed that NPs have the advantage of offering novel structural classes of schistosomicidal drugs because of their well-documented, improved coverage of chemical space relative to large synthetic compounds [17,18,19]. A prominent example of a semi-synthetic NP derivative used against neglected tropical diseases is ivermectin, a semi-synthetic anthelmintic drug derived from monocyclic lactones produced by *Streptomyces avermitilis* [20].

With the automation of analysis methods and organic synthesis, NPs have been given less attention by the pharmaceutical industry for some time [21]. Technologies such as high throughput screening (HTS) and combinatorial chemistry have gained much ground as they promised fast access to novel bioactive compounds [22]. However, because the high expectations raised from automated techniques have not been met, scientists are progressively returning to NP sources for drug discovery and development [23,24]. Currently, several commercial suppliers of NPs allow downloading the structure-data information from their websites. In addition to offering native NPs, some of these vendors also generate NP-based combinatorial structure libraries [25,26,27].

Moreover, the use of computational strategies, such as bioinformatics and chemoinformatics, is expected to accelerate the discovery of novel NPs with schistosomicidal activity, especially in the identification of unexploited, biologically active chemical scaffolds. These computational tools have evolved, aiming at analyzing, understanding, and predicting the bioactivity of novel compounds [28,29]. 

Based on the above considerations, as well as on our continuous interest in NPs and computational strategies, the present review highlights the potential of NPs as sources of prospective leads against schistosomiasis, focusing on recently published findings. Specifically, we focus on compounds of known molecular structure isolated from plants, fungi, bacteria, and marine organisms for which *in vitro* or *in vivo* schistosomicidal activity has been confirmed. Lastly, we propose the currently available computational tools as an alternative and/or complement for discovery of novel NPs with schistosomicidal activity.

## 2. Natural Products as Lead Compounds against Schistosomiasis

In recent years, schistosome research has undergone significant progress, but a new chemical entity approved for schistosomiasis treatment is still absent. Indeed, intense efforts are now directed at the discovery of plant extracts with schistosomicidal activity. However, only a few studies have focused on the isolation, identification, and biological evaluation of NPs from plants, fungi, and other organisms [30]. These NPs have shown potential schistosomicidal lead activity; but, due to the fact that schistosomiasis is a neglected tropical disease only a few NPs have undergone clinical evaluation. 

In the following sub-sections, we will present the major NPs that have shown schistosomicidal activity, paying special attention to those disrupting the pairing of males and females, reducing egg production, interfering with the morphology or constitution of the tegument, motor activity, and the number of dead worms.

Pairing is a fundamental process for schistosome viability inside the human host and for establishing the infection. During pairing, the female is maintained in the gynecophoric canal in the male body in order for sexual maturation and egg production to occur. The induction of separation of males and females reduces or arrests the release of the eggs, which is a relevant factor in the formation of inflammatory granuloma caused by deposited parasite eggs and the transmission of schistosomiasis [31]. Motor activity alterations are also an important indicator of schistosomicidal activity, particularly in those elements of the neuromuscular system that control muscle function and movement. In addition to movement, schistosomes use their neuromuscular systems to control the muscles of the suckers, which allow the worm to attach to the host, the muscle lining of the viscera, including the reproductive, excretory, and digestive tracts, and also the tight coupling of males and females [32,33]. The schistosome tegument plays a crucial role in host-parasite interactions, nutrient uptake, and parasite growth and development, and it helps protect against host immune responses [34]. 

### 2.1. Artemisinins

**Figure 1:**
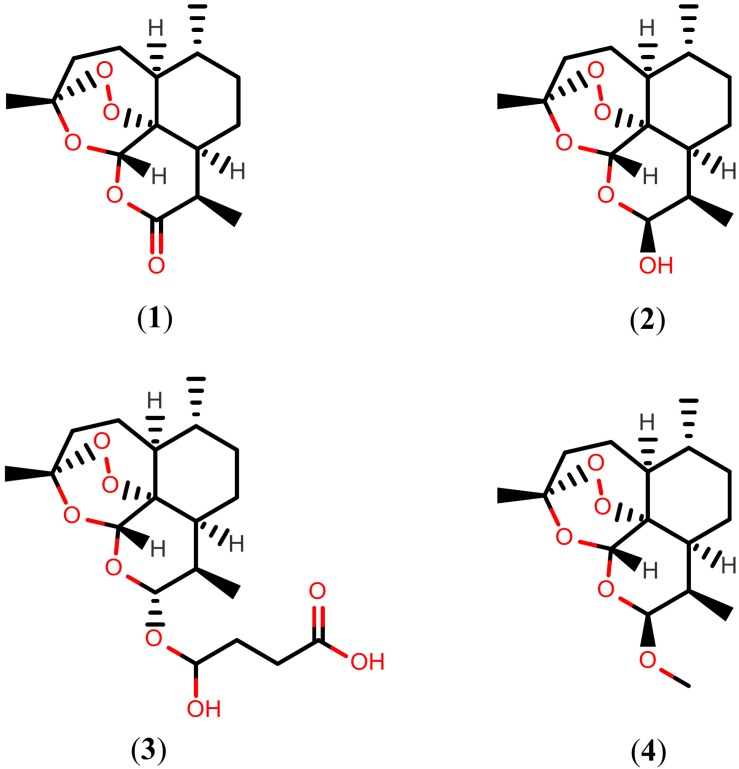
Chemical structures of artemisinins.

Artemisinins are a family of sesquiterpene trioxane lactones derived from the sweet wormwood, (*Artemisia annua*), a medicinal herb that has long been used in Traditional Chinese Medicine to treat malaria. This class of potent anti-malarial compounds was inspired from artemisinin (**1**), which is the parent compound that was isolated in a Chinese drug discovery screen for traditional herbal extracts against *Plasmodium* species in the 1970s [35,36,37]. Importantly, since the 1980s, artemisinins have also been shown to be efficacious against *Schistosoma* species. Treatment of experimentally infected mice with 500 mg/kg of drug **1**, showed significant erosion, peeling, sensory structure damage, and vesicle formation on the tegument of *S. mansoni* 30 days post-infection [38]. However, due to artemisinin’s low solubility, the semi-synthetic drugs dihydroartemisinin (**2**), artesunate (**3**), and artemether (**4**) were subsequently produced. The structures of drug **1**, and its semi-synthetic analogs **2**–**4**, are shown in Figure 1.

The administration of drug **2** in *S. japonicum*-infected mice at a single dose of 300 mg/kg during on days 7 and 35 post-infection reduced the total worm burden by 65% and 61%, respectively, indicating the satisfactory activity of the drug against schistosomula and adult *S. japonicum* worms [39]. An extended study to evaluate the efficacy against *S. mansoni* infected mice using the same dose, showed that drug **2** exhibited best activity at 14–21 days (schistosomula) and 49–56 days (adult worms) post-infection, with corresponding worm reduction rates of 77%–82% and 61%–63%, respectively [40]. 

The schistosomicidal activity of drug **3** was described more than three decades ago [41]. Treatment with drug **3** at a dose 300 mg/kg for two consecutive days showed a prophylactic *S. mansoni* worm reduction rate of 93% in mice at 7–8 days post-infection (schistosomula). However, this drug displayed low efficacy when the mice were treated after day 21, but the effect against the young adult stage remained significant (days 35–36) with a worm reduction rate of 46% [42]. To assess the prophylactic effect of drug **3** against *S. japonicum*, the drug was also given to experimentally infected mice at a single oral dose of 300 mg/kg on each of days 7 and 8 post-infection, resulting in a total worm burden reduction of 73%, while the same treatment given on days 35 and 36 post-infection reduced total worm burden by 69% [43]. However, an *in vivo* study with experimentally infected mice suggested that drug **3** is more effective than PZQ in causing tegumental damage in adult *S. mekongi*, a species found in Cambodia and Laos. Three days after exposure to drug **3**, the tegument of the parasite showed severe swelling, vacuolization, fusion of the tegumental ridges and loss or shortening of the spines on the trabeculae, collapse, and peeling [44]. 

Several studies have also demonstrated the schistosomicidal activity of drug **4**. Treatment of *S. mansoni* infected mice with 400 mg/kg of drug **4** (days 14–21) resulted in a prophylactic worm reduction rate of 75%–82%, while the same treatment given 35 days post-infection reduced total worm burden by 49% [45]. A similar study showed that a dose of 400 mg/kg given on day 49 reduced total worm burden by 55% [46]. The administration of drug **4** at an initial dose of 300 mg/kg on days 14, 21, or 28 post-infection reduced total worm burden by 78%–99% in hamsters infected with juvenile *S. haematobium*, while the same treatment given 77 days post-infection reduced total worm burden by 25% [47]. In addition, further investigation showed post-treatment tegumental alterations, particularly collapse of the oral sucker, extensive swelling, erosion and peeling of tegumental ridges, and destruction of discoid-like sensory structures [48]. Additionally, administration of drug **4** at a single dose of 300 mg/kg during three consecutive days reduced total worm burden by 86% (days 6–8) and 75% (days 34–36) in mice infected with *S. japonicum* [49]. 

Curiously, schistosomules appear to be more sensitive than adult worms, a fact that may be associated with the effects and mechanisms of the action of artemisinins on different developmental stages of schistosomes. Artemisinins react with the iron ions of heme, derived from hemoglobin digestion, to yield reactive oxygen species and chelates [50,51]. In addition, adult worms have the highest level of specific antioxidant enzymes that neutralize reactive oxygen species as compared to schistosomules [52], which may explain why artemisinins are more effective in killing schistosomules than adults under the same treatment protocols. For this reason, several clinical studies have been initiated for repositioning artemisinins as schistosomicidal drugs [53,54]. Indeed, a decreased risk was observed using drug **4** for the prevention of *S. mansoni* and *S. haematobium* infections [55,56]. However, low cure rates were obtained when drug **3** was used in the treatment of chronic infections caused by *S. mansoni* [57,58,59]. In addition, artemisinins often show synergistic therapeutic effects in combination with PZQ in clinical trials, as demonstrated by two meta-analyses [60,61].

### 2.2. Further Terpenoids

**Figure 2:**
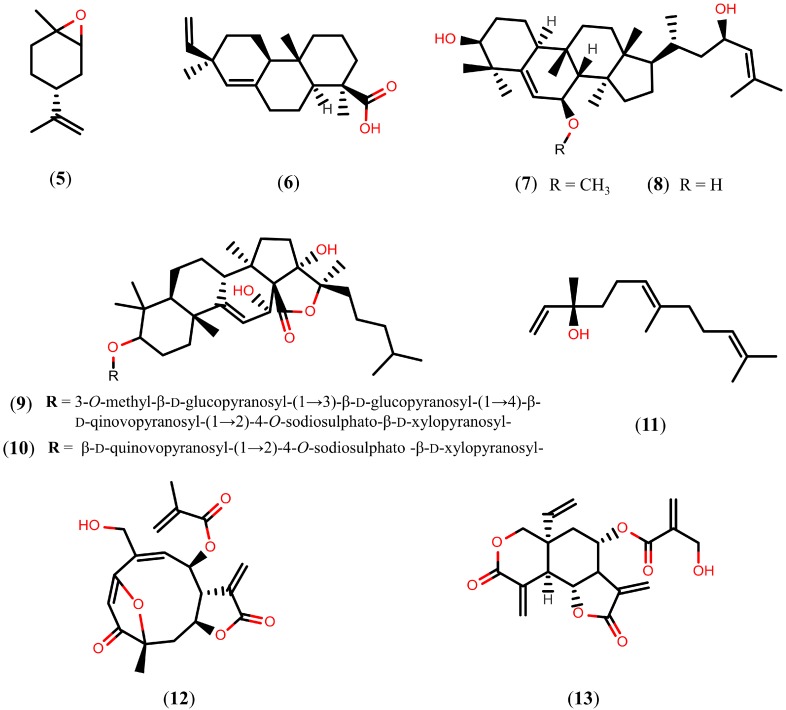
Chemical structures of terpenoids known to have schistosomicidal properties.

Terpenoids are the largest naturally occurring family of hydrocarbons based on combinations of isoprene units that present oxygen functionality in their rearrangements. Terpenoids have a very broad range of biological activities, including anti-malarial [62] and anti-cancer [63,64] properties. In this section, we focus on different types of terpenoids with schistosomicidal activity (Figure 2). First, we refer to (+)-limonene epoxide (**5**), a monoterpene compound isolated from the essential oil of *Citrus sinensis*. *In vitro* studies have demonstrated that, 120 h after exposure to compound **5** at a concentration of 164 µM, all adult *S. mansoni* worms were killed, and complete separation of paired worms occurred. In addition, compound **5** caused moderate tegumental disruption in worms exposed to a concentration of 164 µM, whereas severely damaged worms were seen at higher concentrations (328 µM) [65].

An *in vitro* study with pimaradienoic acid (**6**), a major diterpene isolated from *Viguiera arenaria*, promoted morphological alterations of the *S. mansoni* tegument and caused the separation of 100% of the coupled adult worms into individual male and female worms at a concentration of 100 µM after 24 h of exposure, thus justifying the absence of egg production at this concentration [66]. A preliminary *in vivo* study with experimentally infected mice also indicated that, at a dose of 100 mg/kg/day, compound **6** reduced the total number of adult *S. mansoni* worms by about 40%. Moreover, compound **6** was devoid of *in vitro* cytotoxicity against a human fibroblast cell line at a broad concentration range (7.8–500 µM), indicating its selectivity [66].

The cucurbitane-type triterpenes karavilagenin C (**7**) and balsaminol F (**8**), isolated from aerial parts of *Momordica balsamina*, a plant used in African traditional medicine to treat malaria [67], also exhibited schistosomicidal activity. An *in vitro* study with compounds **7** and **8**, at 50 µM and 100 µM concentrations, respectively, caused the death of 100% of *S. mansoni* adult worms after 24 h of exposure. At 10–50 µM concentrations, both compounds **7** and **8** caused significant reductions in the motor activity of adult worms and significantly decreased egg production. The same study found that all of the paired adult worms were separated into individual male and female worms after 24 h of incubation with 50 µM of compound **7** and 25 µM of compound **8** after 24 h of exposure [68]. However, compound **8** showed significant cytotoxicity against human MCF7 cells, with an IC_50_ value of 16.7 µM [69], while compound **8** showed cytotoxicity against Huh-7 cells and mouse primary hepatocytes at 15 μM [70]. On the other hand, echinoside A (**9**) and echinoside B (**10**), triterpene glycosides isolated from sea cucumbers *Actinopyga echinites* and *Holothuria polii*, respectively, exhibit *in vitro* activity against *S. mansoni* with LC_50_ values of 0.17 µM and 0.35 µM, respectively.

An *in vitro* study with nerolidol (**11**), an aliphatic sesquiterpene isolated from *Baccharis dracunculifolia* [71], reduced worm motor activity and caused the death of all male and female *S. mansoni* adult worms after 96 h of exposure, at concentrations of 31.2 μM and 62.5 μM, respectively. In addition, concentrations higher than 62.5 μM caused morphological alterations on the tegument of worms, such as disintegration, sloughing, and erosion of the surface; moreover, a correlation between viability and tegumental damage was observed [72].

The *in vitro* study with goyazensolide (**12**), a sesquiterpene lactone isolated from *Eremanthus goyazensis*, caused the death of 90% of adult *S. mansoni* worms at concentrations up to 5.5 µM in the first 72 h of exposure. For compound **12**, concentrations higher than 2.2 µM resulted in a dramatic reduction of egg production that was probably due to the reduced motility of the worms, a requirement for male and female mating. It was also observed that the separated worms were more susceptible to compound **12** than the pairing worms, and the female worms alone were significantly more susceptible than the male worms [73]. Another sesquiterpene lactone with promising schistosomicidal activity is vernodalin (**13**), a compound isolated from *Vernonia amygdalina*. An *in vitro* study with compound **13** showed complete reduction of the worm’s motor activity and egg-laying of *S. japonicum* adult worms after 24 h of exposure, at a concentration of 55.5 μM. However, a preliminary *in vivo* study with experimentally infected mice indicated that compound **13**, at a non-lethal dose of 60 mg/kg, had no great effect on the parasite [74].

#### Tanshinones

**Figure 3:**
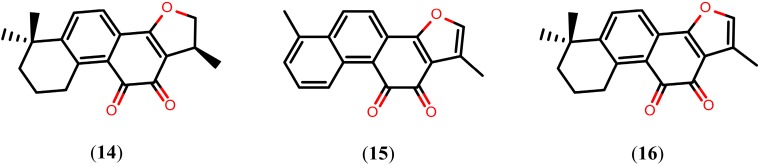
Chemical structures of tanshinones.

The above documented compounds were mainly evaluated based on the number of dead worms and alterations in the tegument after treatment. However, few studies have reported on their mechanism of action. Now that the complete genome sequences of *S. mansoni* [75] and *S. japonicum* [76] have been elucidated, genomic and proteomic approaches can be used to identify potential targets for drug design. In recent years, several interesting drug targets have been proposed, including enzymes that play an important role in the biochemical pathways of schistosomes [77,78,79,80,81]. Tanshinones, a group of abietane diterpenes isolated form *Salvia miltiorrhiza* and originally considered for the prevention and treatment of cardiovascular and cerebrovascular diseases [82,83], have been shown to inhibit *S. mansoni* thioredoxin glutathione reductase (*Sm*TGR). These compounds, more specifically cryptotanshinone (**14**), tanshinone I (**15**), and tanshinone IIA (**16**), inhibit the *Sm*TGR enzyme with IC_50_ values of 3.9 µM, 8.9 µM, and 10.9 µM, respectively [84]. Structures of the tanshinones (compounds **14**–**16**) are shown in Figure 3. Schistosomes possess a streamlined thiol-based redox system in which a single enzyme, *Sm*TGR, a fusion of a glutaredoxin domain to canonical thioredoxin reductase domains, supplies electrons to oxidized glutathione and thioredoxin. Therefore, after inhibition of *Sm*TGR, schistosomes die of oxidative damage from the host, such as lipid peroxidation, and protein and DNA oxidation, thereby suggesting that tanshinones can be promising leads for drug design [85,86,87].

### 2.3. Alkaloids

**Figure 4:**
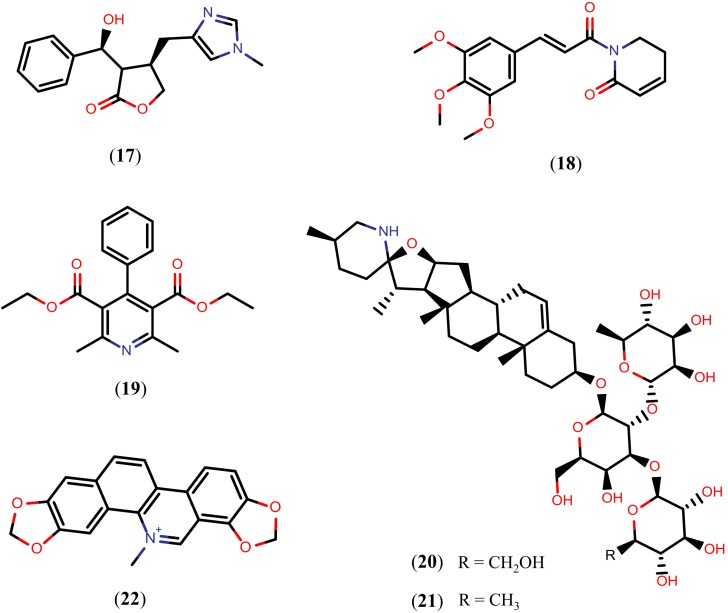
Chemical structures of alkaloids known to have schistosomicidal properties.

Alkaloids are a group of NPs that contain basic nitrogen atoms. They are widely found in plants, bacteria, fungi, and animals. Several alkaloids have schistosomicidal activity, many of which display promising biological activities (Figure 4). The imidazole alkaloid epiisopiloturine (**17**), isolated from the leaves of *Pilocarpus microphyllus*, caused extensive disruption of tegument, such as sloughing, total reduction of motor activity, and the death of all *S. mansoni* adult worms 120 h post-exposure *in vitro* (at 523 µM). Using the sub-lethal concentration of 350 µM, compound **17** caused a 100% reduction in egg production. In addition, compound **17** showed selective schistosomicidal activity and exhibited lower cytotoxicity to Vero mammalian cells (1.7 mM had no noticeable effect on cell viability) [88].

Piplartine (**18**), an amide alkaloid found in several *Piper* species such as *P. tuberculatum*, decreased motor activity and caused the death of all adult *S. mansoni* worms (at 15.8 µM concentration) within 24 h of *in vitro* exposure. Additionally, compound **18** caused a 75% reduction in egg production (sub-lethal concentration of 6.3 µM) and showed lower cytotoxicity to Vero cells after treatment with the maximum concentration tested (at 31.5 µM) [89].

Diethyl 2,6-dimethyl-4-phenylpyridine-3,5-dicarboxylate (**19**), a pyridine alkaloid isolated from the rhizome of *Jatropha elliptica*, caused complete motor activity reduction and the death of 100% of adult *S. mansoni* worms (at 152 μM) after 96 h of *in vitro* exposure. Under the same concentration, compound **19** also caused the separation of all paired worms and extensive disruption of their tegument through sloughing, formation of vesicles, and vacuolization. Curiously, at a concentration of 12.2 μM, compound **19** completely immobilized cercariae after 30 min of exposure, with a value of LC_100_ = 6.1 μM [90]. Importantly, visual comparison of the chemical structure of compound **19** with nifedipine, a Ca^2+^ channel blocker active against schistosomules and adult worms of *S. mansoni*, suggests that these compounds share the same mechanism of action [91]. 

Similar *in vitro* effects were produced by solasonine (**20**) and solamargine (**21**), two glycoalkaloids isolated from fruits of *Solanum lycocarpum*. Compounds **20** and **21**, at a 50 µM concentration, caused the death of all *S. mansoni* adult worms, complete separation of all pairing worms, and extensive disruption of their teguments through sloughing, as well as death, within 24 h of exposure. However, it was observed that these glycoalkaloids did not significantly reduce the number of eggs laid by the adult worms [92]. 

On the other hand, an *in vitro* study with sanguinarine (**22**), an enzylisoquinoline alkaloid isolated from *Sanguinaria canadensis*, at a concentration of 10 µM, resulted in 100% mortality of adult *S. mansoni* worms 48 h post-exposure and caused severe erosion and disintegration of the tegumental surface between tubercles. Interestingly, compound **22** also exhibited the strongest cercaricidal activity *in vitro*, killing all cercariae at a concentration of 0.5 μM after an exposure time of two hours [93].

#### Quinoline Methanols

**Figure 5:**
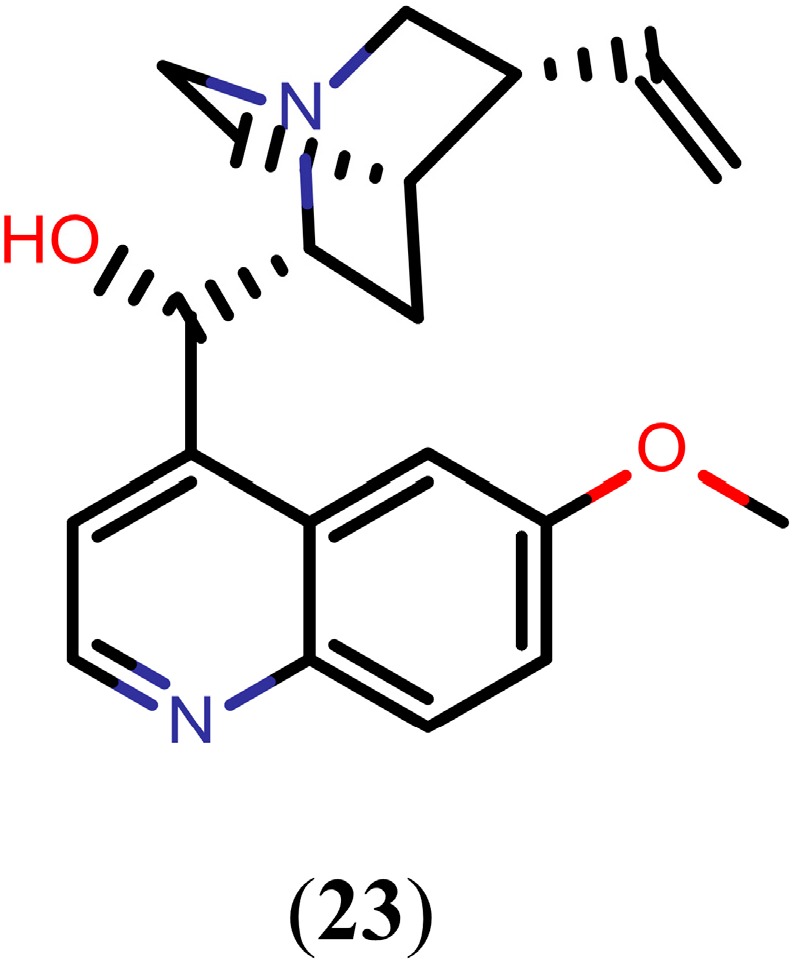
Chemical structure of quinidine.

Quinoline methanols are alkaloids with a methanol group at the 4-position of the quinoline scaffold. Since 1811, when the first of these quinolines was isolated from *Cinchona officinalis*, many other quinoline methanols have been isolated and widely used for the treatment of malaria. Remarkably, quinoline methanols, such as quinidine (**23**, Figure 5), have also been shown to exhibit promising *in vivo* activity against *S. mansoni* worms. Drug **23**, at a dose of 75 mg/kg/day, caused 61% decrease in worm burden and 54%, 72%, and 98% decrease of egg production in the liver, small intestine, and large intestine, respectively [94]. 

Currently, the exact mechanism of action of quinoline methanols in schistosomes is not fully understood, although interference with hemoglobin digestion seems to play a role, as observed in malaria parasites. Digestion of hemoglobin provides adult schistosomes with essential nutrients, such as amino acids, for growth and reproduction. However, during this process schistosomes are forced to convert toxic heme into a nontoxic hemozoin (Hz). Adult females of *S. mansoni* produce large amounts of pigment known as Hz within the gut [95]. It has been demonstrated that drug **23** inhibited the formation of Hz in *S. mansoni* female homogenates (65%) [94]. According to Oliveira and colleagues [96], Hz represents a major heme detoxification mechanism in *S. mansoni*, acting as a preventive antioxidant defense against reactive oxygen species formation, lipid peroxidation, and protein and DNA oxidation. These characteristics indicate that interfering with Hz formation in schistosomes is a valuable approach for drug design.

It is worth mentioning that mefloquine, a synthetic quinoline methanol approved for malaria treatment, is undergoing detailed *in vitro*, *in vivo*, and clinical investigation for its schistosomicidal properties. It is active against all three major *Schistosoma* species and against both the juvenile and adult stages, a characteristic that neither PZQ nor the artemisinins possess [57,97,98,99,100]. Initial results from clinical trials were also promising; the combination of mefloquine and artesunate (**21**) achieved an egg reduction rate of 95% against *S. haematobium* in school-aged children [57]. However, combination therapy with mefloquine and PZQ, which showed high worm burden reductions in laboratory animals, has not yet been studied in *Schistosoma*-infected humans [101].

### 2.4. Flavonoids

**Figure 6:**
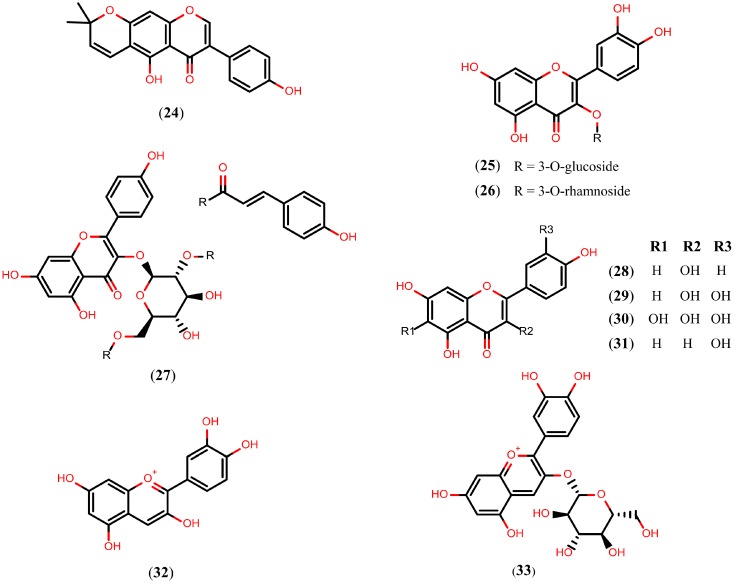
Chemical structures of flavonoids known to have schistosomicidal properties (compounds **24**–**28**) and *Sm*NACE inhibitors (compounds **29**–**33**).

Flavonoids are a chemical class of plant secondary metabolites that are derivatives of 2-phenyl-benzyl-γ-pyrone. Over 9000 flavonoids of different subgroups are known. The majority of flavonoids are present in the glycosylated form under natural conditions [102,103]. Because of their diverse chemical structure and the variety resulting from the attached substituents, flavonoids have been proposed to exert beneficial effects on a multitude of diseases [104,105,106] including schistosomiasis. The structures of flavonoids reported to display schistosomicidal activity (compounds **24**–**28**) are shown in Figure 6.

The alpinum isoflavone (**24**), an isoflavone isolated from *Millettia thonningii*, exhibited cercaricidal activity *in vitro*, killing all cercariae at a concentration of 150 μM after 30 minutes of exposure. Moreover, compound **24**, at a concentration of 75 µM, also killed 90% of the adult *S. mansoni* worms after 72 h of exposure and caused 100% of the decrease in egg production [107]. Isoquercetin (quercetin 3-*O*-glucoside) (**25**), a flavonol isolated from the aerial parts of *Roupala montana*, caused 75% of the decrease in motor activity of adult *S. mansoni* worms incubated *in vitro* under a concentration of 100 µM. Quercitrin (**26**), a similar flavonol isolated from the aerial parts of *Roupala montana* and *Schefflera vinosa*, also caused 75% of the decrease in motor activity at a 50 µM concentration [108]. 

Kaempferol-3-*O*-(2'',6''-di-*O*-(*E*)-*p*-coumaroyl)-β-d-glucopyranoside (**27**) and kaempferol (**28**), flavonols isolated from the aerial parts of *Styrax pohlii* and *Styrax camporum*, respectively, triggered the separation of all paired adult *S. mansoni* worms incubated *in vitro* at a 100 µM concentration. Compounds **27** and **28** also exhibited *in vitro* IC_50_ values of 35.5 µM and 25 µM regarding the pairing of *S. mansoni* , respectively [109]. Thus, while most of the flavonoids were not able to kill the worms, they exhibited significant reduction in motor activity or pairing of the *S. mansoni* adult worms.

The mechanism of action by which compounds **24**–**28** exert their schistosomicidal activity is unclear. However, quercetin (**29**), a flavonol also isolated from *S. camporum* that moderately reduces *in vitro* motor activity (100 μM) [109], was identified as a selective inhibitor of the *S. mansoni* NAD^+^ catabolizing enzyme (*Sm*NACE) at an IC_50_ value of 3.9 µM. *Sm*NACE is an important target localized to the outer tegument of the adult parasite and it is presumably involved in the parasite’s survival by manipulating the host’s immune regulatory pathways. The discovery of flavonoids that inhibit *Sm*NACE in the low micromolar range has led to considering flavonoids as promising drug candidates for treating schistosomiasis. Different flavonoids, more specifically quercetagetin (**30**), luteolin (**31**), cyanidin (**32**), and kuromanin (**33**), inhibit the *Sm*NACE with IC_50_ values of 1.3 µM, 8.4 µM, 2.3 µM, and 8.2 µM, respectively (Figure 6) [110].

### 2.5. Arachidonic Acid

**Figure 7:**
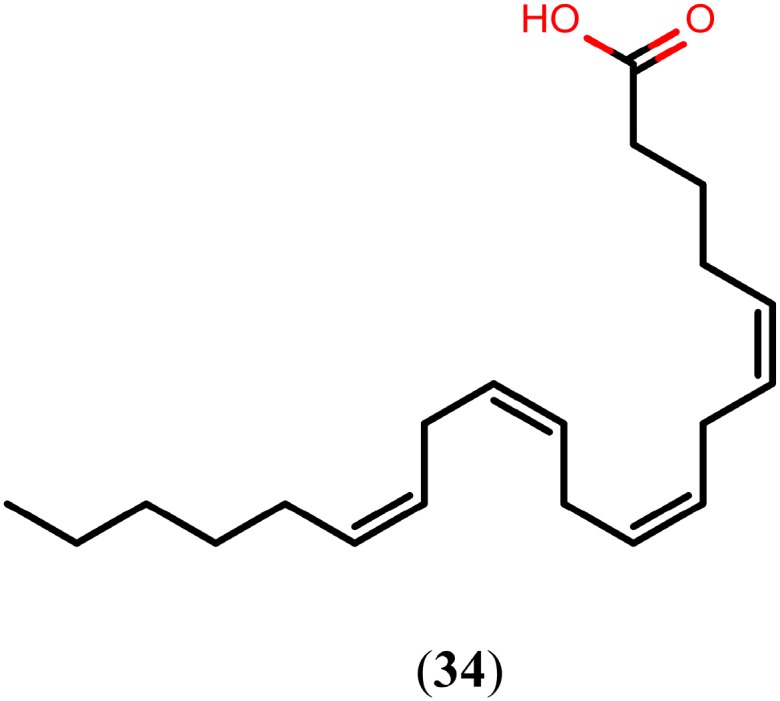
Chemical structure of arachidonic acid.

Arachidonic acid (**34**, Figure 7) is a long-chain essential polyunsaturated fatty acid belonging to the omega-6 group. It plays an important role in metabolic processes as a precursor of prostaglandins, leukotrienes, and thromboxanes. Due to a growing interest in the application of compound **34** in various fields of dietary and health requirements, much attention has been given to the industrial production of **34**-containing oil through the cultivation of *Mortierella alpina* [111,112,113]. Recently it has been shown that compound **34** killed all juvenile and adult schistosomes *in vitro* at a concentration of 10 mM and displayed significantly high worm burden reduction (63%–81%) in mice and hamsters infected with *S. mansoni* and *S. haematobium*, respectively, at a dose of 1000 mg/kg. In addition, *in vitro* exposure to 5 mM of compound **34** caused extensive damage, disorganization, and degeneration of the tegument and of the subtegumental musculature [114]. 

Compound **34**-mediated killing is essentially due to excessive activation of schistosome magnesium-dependent neutral sphingomyelinase, leading to the hydrolysis of sphingomyelin to ceramide and phosphorylcholine [114]. Consequently, sphingomyelin hydrolysis elicits an increase in membrane permeability, bending, and aggregation as well as dramatic perturbations in the lipid content and rigidity of the schistosome, allowing the free movement of antigenic molecules along the plane of the surface membranes and avid binding of specific antibodies [115,116]. For this reason, clinical studies were initiated in children for the development of **34** as a safe and cost-effective schistosomicidal drug, especially because no related adverse effects of the drug were recorded in the *in vivo* experiments [117].

### 2.6. Quinones

**Figure 8:**
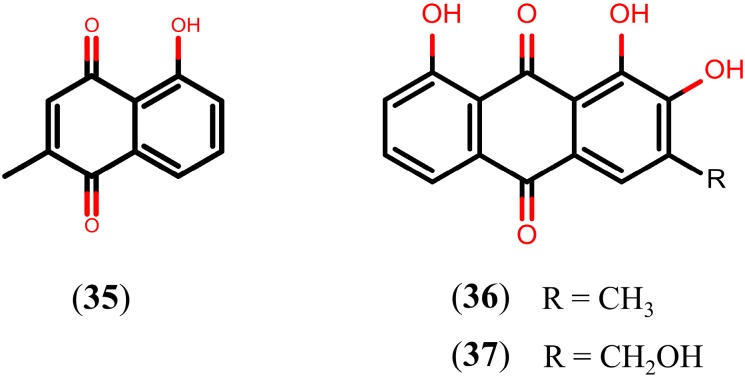
Chemical structures of quinones.

Quinones are a class of natural oxidized derivatives of aromatic compounds with the quinone structure and they can be mainly classified into three types—benzoquinone, naphthoquinone, and anthraquinone—according to the number of benzene rings in the structural skeleton and the presence of fused rings [118]. So far, three quinones have been reported as leading candidates in schistosome discovery (Figure 8). The *in vitro* study with plumbagin (**35**), a naphthoquinone isolated from the aerial parts of *Plumbago scandens* [119], resulted in 100% mortality of adult *S. mansoni* worms at 48 h post-exposure and caused severe erosion and disintegration of the tegumental surface between tubercles, at a concentration of 10 µM. In the same study, it was also found that the exposure of adult *S. mansoni* worms, at a 10 µM concentration of compound **35** for 24 h, resulted in prominent alterations of the tegumental surfaces, usually with disintegration of tubercles and often accompanied by a decrease in the number of spines. In addition, compound **35** also exhibited strong cercaricidal activity *in vitro*, killing all cercariae, at a concentration of 0.5 μM, after an exposure time of three hours [93].

An *in vitro* study with norobtusifolin (**36**) and kwanzoquinone E (**37**), anthraquinones isolated from roots of *Hemerocallis fulva*, exhibited significant activity by completely immobilizing all *S. mansoni* cercariae after an exposure time of 15 s (at 11.4 µM) and 12 min (at 87 µM), respectively. In the same study, motor activity of all *S. mansoni* adult worms was also interrupted within 16 h by compound **36** (at 185 µM) and compound **37** (at 175 µM). Following removal of the compounds, 35% and 55% of the adults exposed to these compounds, respectively, were found to be dead. In contrast to the effects on the cercariae and adults, none of these compounds displayed activity on the schistosomula stage [120].

### 2.7. Other Natural Products

**Figure 9:**
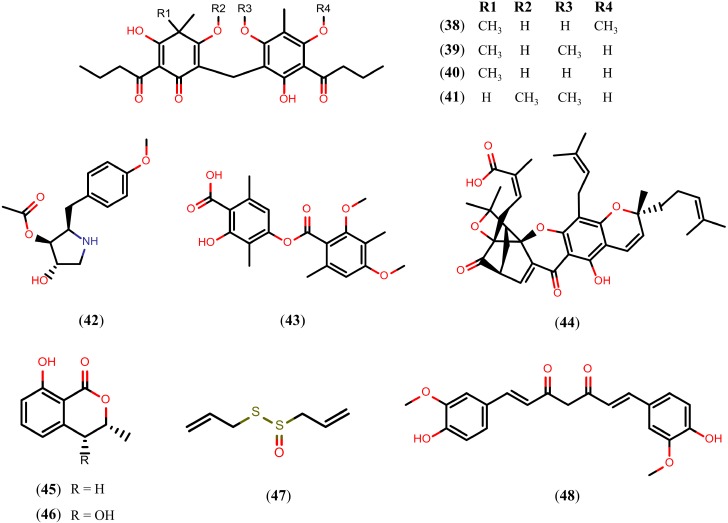
Chemical structures of other NPs known to have schistosomicidal properties.

A variety of NPs belonging to the alkaloid, terpenoid, and flavonoid families present schistosomicidal activity. However, schistosomicidal activity of the NPs is not just limited to these classes. Figure 9 presents NPs belonging to other chemical classes or other natural sources with schistosomicidal activity. First, we refer to phloroglucinol derivatives, a distinct class of NPs obtained from the rhizomes of *Dryopteris* species that showed satisfactory activity against *S. mansoni* adult worms. *In vitro* studies showed that after 24 h of exposure to aspidin (**38**) and desaspidin (**39**) (25 μM), and flavaspidic acid (**40**) and methylene-bis-aspidinol (**41**) (50 μM and 100 μM, respectively), all of the adult worms were eliminated. All of the paired adult worms were also separated into individual male worms and female worms after 24 h of exposure to compound **41** (10 μM), and compound **40** (50 μM). In addition, all of the worms exposed to compounds **38** and **39** showed decrease motor activity at 10 μM concentration, while exposure to compounds **40** and **41** caused decrease motor activity at 25 μM and 100 μM concentrations, respectively [121].

Antimicrobial agent anisomycin (**42**), produced by *Streptomyces griseolus*, quickly kill (within hours) all schistosomules and adult *S. mansoni* worms *in vitro* at a 1 μM concentration. Similar effects were produced by diffractaic acid (**43**), a depside compound that can be isolated from *Usnea longissima*, and gambogic acid (**44**), a xanthone isolated from dry latex of *Garcinia hanburyi* [122]. 

Dihydroisocoumarins, such as *R*-(−)-mellein (**45**) and *cis*-(3*R*,4*R*)-4-hydroxymellein (**46**), isolated from the fungus *Apiospora montagnei*, also showed *in vitro* activity against *S. mansoni* adult worms. Despite the high similarity between their chemical structures, compounds **45** and **46** caused the death of 100% of the parasites (both male and female) at 1.1 mM and 257 μM concentrations, respectively. In addition, after 120 h of incubation, compound **45** (280 μM) caused the separation of 100% of the paired adult worms and extensive damage and severe disruption of tegument at the 1.1 mM concentration). Compound **46** had no effect on the worm pairing of *S. mansoni* when tested in concentrations of up to 257 μM [123].

An *in vitro* study with allicin (**47**), the main constituent of *Allium sativum*, showed wrinkling of the tegument of *S. mansoni* adult worms (at 30 mM concentration), while a concentration of 60 mM resulted in damage to tubercles, and the thorns became shorter and fewer. With a 90 mM concentration, compound **47** caused an extensive disruption of the tegument through the formation of vesicles and ulcers in the worm’s musculature.

Lastly, curcumin (**48**), a diarylheptanoid isolated from the rhizomes of *Curcuma longa*, was shown to cause the death of all adult *S. mansoni* worms *in vitro* and it inhibited egg production by 100% at a 50 μM concentration. In addition, worms exposed to compound **48** (5–20 μM) showed decreased motor activity without tegumental alterations [124]. The mechanism by which compound **48** exerts its schistosomicidal activity is not clear. However, it has been proteasome proteolytic and cellular deubiquitinating activities [125,126]. Therefore, the schistosomicidal activity of compound **48** may be due to the inhibition of the ubiquitin-proteasome pathway of the *S. mansoni*.

## 3. Drawbacks in NP Lead Discovery

The discovery of NPs with schistosomicidal activity is a complex process that is intimately linked to the time and costs involved in the isolation and structure elucidation of compounds and biological assays [127,128,129,130]. The isolation of NPs combines various separation techniques that depend on the solubility, volatility, and stability of the compounds that are to be separated. Taking into consideration that a plant, marine organism, or microorganism may contain thousands of constituents, the separation and isolation process can be long and tedious. Furthermore, evaluation of schistosomicidal activity should be done after the purification process in order to exclude interference from the accompanying NPs [127,128].

Current phenotypic methods that are utilized to assess schistosomicidal activity involve visual interpretation. Microscopy techniques for analysis of parasite viability are currently the gold-standard *in vitro* assays to determine schistosomicidal effects. In this context, morphological changes in the tegument and motility reduction are visualized and schistosome viability is defined by the percentage of motile live parasites or by using a predetermined scale based on motility and worm morphology [129,130]. However, because of the lack of automatization of this process the evaluation of a large number of compounds is not always practicable. 

In an effort to solve this problem, the Alamar blue (AB) assay has been proposed as an alternative to assess schistosomicidal activity. AB is an oxidant agent used as an indicator of antioxidant activity and cell viability, and it can be measured rapidly and automatically by fluorescence. This assay can provide objective readouts of death rates in juvenile and adult schistosomes and it is amenable to automation for HTS. However, there are several drawbacks to using AB, such as the possibility of damage to juvenile worms due to low expression of antioxidant enzymes, and it is less reliable than microscopy for more subtle morphological changes including those induced by some known schistosomicidal drugs [131].

Since visual assessment of schistosome viability is time-consuming, novel HTS techniques have recently been developed in order to automate schistosome viability analysis [122,132,133]. Recently, high-content screening has been developed using microscopes to capture images of the juvenile schistosomes in order to assess motility changes. Indeed, bright-field imaging can simultaneously provide data on many phenotypic parameters (e.g., size, shape, granularity, vacuolation, tegumental damage, motility). However, high-content screening machines that produce large amounts of data depend on an extensive mathematical modeling for correct scoring of motility using the captured images [134,135,136,137].

In addition, the access to adequate quantities and a steady source of whole schistosomes is a recurring concern at compound screening labs. With increased demand for *in vitro* assays, there is also a corresponding increase in life cycle maintenance costs as well as additional costs related to the breeding of snail hosts and to the care of the experimental animals [130].

## 4. Integration of Natural Products and Virtual Screening

One sophisticated strategy that can accelerate the discovery of NPs with schistosomicidal activity is virtual screening (VS), which is defined as the use of computational filters in a database of chemical structures to predict the bioactivity of a compound with respect to a specific target. The major advantage obtained from VS is the reduction in time and resources required for an *in vitro* screen of a chemical library of known compounds. Therefore, by predicting inactive compounds in a VS, the number of compounds to be tested *in vitro* can be dramatically reduced, sometimes by orders of magnitude. Due to elimination of inactive compounds, the hit rates in the *in vitro* assays are often much higher compared to HCS or random *in vitro* testing without preliminary VS [28,29,138]. Currently, several strategies exist that can be applied to the identification of novel schistosomicidal lead compounds using VS methods. However, due the fact that schistosomiasis is a neglected tropical disease, the use of VS is still poorly explored, although some examples have appeared recently in the literature [139,140,141,142]. 

VS is a process that can be divided into several well-defined steps. The VS process begins with the design of the experiment, and the availability of active compounds or three dimensional (3D) specific targets (e.g., enzymes, receptors, ion channels) will indicate which computational strategy should be used. Then, a comprehensive validation and optimization of the VS models is performed using appropriate metrics that are highly recommended to guarantee that the models are able to distinguish between active and inactive compounds. Lastly, VS proceeds using a virtual database of compounds and the best validated and selected models [143]. 

Indeed, the basic requirement for NP-based discovery using VS is a searchable structure database of NPs [28]. Several NP databases are freely available for VS use, such as NuBBE [144], Super Natural [145], the Database of Traditional Chinese Medicine [146], and the Seaweed Metabolite Database [147]. A summary of some of the available NP databases is presented in Table 1. Some of these databases are commercial suppliers of NPs that offer the ability to download structure-data files from their corporate homepages. However, this initiative may also make use of the compilation of scientific papers containing information on pure NPs. In this step, researchers must keep in mind that the use of non-commercial databases may introduce an important problem: the NP will have had to have been isolated for experimental evaluation.

Although in-depth discussion of VS methods and NPs databases are beyond the scope of this current review, we discuss the main VS strategies that can be used to identify novel schistosomicidal lead compounds: ligand-based virtual screening (LBVS) and structure-based virtual screening (SBVS) [148,149].

**Table 1:** Some available NPs databases for virtual screening.

Database Name	Nº of Entries	Website
NuBBE Database	643	[144]
Traditional Chinese Medicine Database	>32,300	[150,151]
Dictionary of Natural Products	>260,000	[152]
Specs Natural Products	400	[27]
Herbal Ingredients’ Targets Database	586	[153]
Super Natural Database	>325,500	[145]
NPACT Database	1574	[154]
Database of Indonesian Medicinal Plants	6776	[155]
Greenpharma Natural Compound Library	>150,000	[156]
Tea Metabolome Database	1450	[157]
TimTec Natural Products Library	720	[25]
Seaweed Metabolite Database	1055	[147]
InterBioScreen Natural Products Database	>17,500	[26]
MAPS Database	>1200	[158]
Database of Traditional Chinese Medicine	>12,000	[146]
TIP Database	8856	[159,160]
AMRI’s Natural Product Library	>290,000	[161]

### 4.1. Ligand-Based Virtual Screening

Ligand-based virtual screening (LBVS) is the strategy of choice when the biological target is not known or its 3D structure is not available. In this approach, structure-activity data from a set of known schistosomicidal compounds can be used to generate the models. The most widely known LBVS methods are ligand-based pharmacophore models and machine learning techniques [149]. According to the International Union of Pure and Applied Chemistry (IUPAC), a pharmacophore is the ensemble of steric and electronic features that is necessary to ensure the optimal supramolecular interactions with a specific biological target structure and to trigger (or to block) its biological response [162].

**Figure 10:**
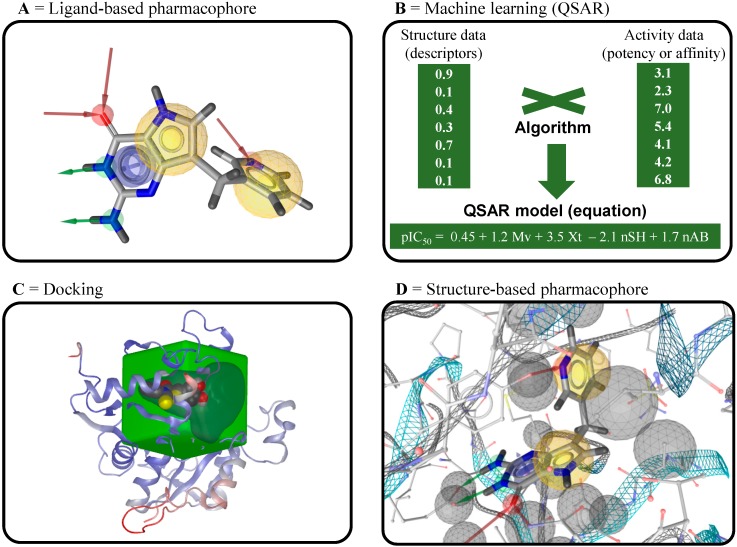
Ligand- and structure-based tools used in virtual screening. (**A**,**D**) Representative pharmacophoric points used for construction of ligand- and structure-based pharmacophore models constructed using the LigandScout program. Hydrogen bond donors and acceptors are represented as green and red vectors, respectively; aromatic PI-interactions are represented by blue planes; lipophilic areas are represented as a set of yellow spheres; and steric constraints are represented by gray spheres; (**B**) Summary of the development of QSAR models from a set of known active and inactive compounds; (**C**) Representation of 3D coordinates of a binding site used for docking calculations in the OEDocking program.

In VS, ligand-based pharmacophores identify key common pharmacophoric points and the relative orientations of known active compounds that are not shared by the inactive compounds. Figure 10A presents the most common pharmacophoric points used with filters in ligand-based VS. Therefore, the use of these models in a subsequent search involves the retrieval of compounds in virtual databases that contain pharmacophoric points similar to known active compounds. Several programs for pharmacophore modeling are widely used, including commercially available programs such as Catalyst [163], GALAHAD [164], HipHop [165], and PHASE [166]. However, conformational flexibility of known active compounds represents one of the main bottlenecks in pharmacophore model generation, because the bioactive conformations of these compounds are usually not known.

Machine learning methods are used to develop statistical models from a training set of known active and inactive compounds (Figure 10B). One of the most important characteristics of machine learning models is their predictive power. In the last decade, supervised learning has been widely used for the development of quantitative structure–activity relationship models (QSAR) and then applied to untested chemical compounds for the numerical prediction of biological activity (continuous models), discrimination between active/partially active/inactive compounds (multiclass models), and discrimination between active/inactive compounds (binary models) [167,168]. To construct QSAR models, molecular descriptors are calculated for all compounds with biological data for a specific endpoint. These descriptors are used to train the algorithm. Once validated, the generated models, constitute the starting point for the activity prediction of new compounds in VS [169,170,171,172]. The methods of validation for these QSAR models have been widely-discussed in the literature (see references [171,173,174,175,176,177,178]).

### 4.2. Structure-Based Virtual Screening 

Structure-based virtual screening (SBVS) explores information about a 3D structure of targets that are either determined experimentally by, for example, X-ray crystallography and nuclear magnetic resonance, or are computationally predicted through homology modeling in order to select ligands that are likely to interact favorably. Because of the advances in genomics and proteomics, several 3D structures of schistosome targets have been solved and stored in public databases, such as the RCSB Protein Data Bank (PDB). However, because of its knowledge-based feature, SBVS strongly depends on the amount and the quality of information available about the system under investigation. No matter what kind of 3D protein is employed as a target, important issues must be properly addressed, including the consideration of the water molecules in the binding site, the X-ray resolution, the flexibility of the target, the selection of the most relevant geometry, and a suitable assignment of the protonation and tautomeric states [179,180,181].

In the past few years, several docking programs, such as OEDocking [182,183,184], CovDock [185], FlexX [186], and Gold [187], have been proposed for SBVS. Docking involves fitting the ligand into the binding site of a 3D target in order to predict the binding affinity followed by the generation of a ranking of the fit using scoring functions (Figure 10C) [188]. Most docking programs incorporate ligand flexibility into the docking calculations so that the binding geometry of the ligand can be correctly predicted. However, the 3D structure of the target is usually assumed to be mostly rigid, as the explicit inclusion of target flexibility in the docking calculations would be too computationally demanding [189]. In addition, despite numerous published papers demonstrating the use of docking for VS, it still remains a major challenge because the empirical scoring functions have been found to have limited accuracy in the ranking of compounds, and it is not always possible to distinguish between active and inactive compounds. Therefore, the use of several different scoring functions, followed by the fusion of the scores to create a consensus score, is a highly advisable approach [190,191,192].

Another SBVS strategy is the development of structure-based pharmacophore models, which can be generated directly from the complex binding site and a ligand of the 3D target (Figure 10D). Some programs, such as LigandScout [193], allow for the complete exploration of the intermolecular interactions of a ligand into the binding site of a 3D target and the inclusion of shape and volume information derived directly from the structural data. This strategy determines the chemical features based on complementarities between a ligand and its binding site. In this case, steric constraints in the form of inclusion volume are added to make sure that the lipophilic parts of the ligand are matched correctly with the binding site in the VS run. Indeed, the purpose of the structure-based pharmacophore strategy is similar to the purpose of docking, including the same level of information, but it is less demanding with respect to computational resources [194,195].

### 4.3. Inverse Virtual Screening

In contrast to conventional VS strategies, the aim of inverse VS, also known as target fishing, is to identify the most likely targets of a known bioactive compound. In this innovative *in silico* approach, active compounds without a known mechanism of action are screened against a panel of targets in order to obtain a restricted group of promising targets. Therefore, we suggest that this approach is also potentially applicable to all NPs with known schistosomicidal activity, such as those reported in previous sections, to accelerate structure optimization towards more effective and less toxic semi-synthetic derivatives through a virtual method for a subsequent experimental study. In this context, docking-based methods may be used to elucidate the probable schistosome targets for these NPs based on docking against a panel of 3D targets available in PDB. Finally, scoring functions, which ideally correlate with the free energy of binding, may be used to select possible targets for each NP [196,197,198].
